# A case series of ultrasound and pathological assessment of follicular thyroid tumors: Addressing indeterminate malignancy

**DOI:** 10.1097/MD.0000000000041196

**Published:** 2025-01-17

**Authors:** Xian Wang, Hui Zhou, Muhammad Asad Iqbal, Donggang Pan, Junbo Zuo, Nida Fatima Moazzam, Jin Zhang, Hui Sun

**Affiliations:** a Department of Ultrasound, Affiliated People’s Hospital of Jiangsu University, Zhenjiang, China; b School of Medicine, Jiangsu University, Zhenjiang, Jiangsu, China; c Department of Radiology, Affiliated People’s Hospital of Jiangsu University, Zhenjiang, China; d Department of General Surgery, Affiliated People’s Hospital of Jiangsu University, Zhenjiang, China; e Department of Pathology, Affiliated People’s Hospital of Jiangsu University, Zhenjiang, China.

**Keywords:** follicular, malignant potential undetermined, thyroid gland, ultrasonic imaging

## Abstract

**Rationale:**

Follicular tumors with uncertain malignant potential of the thyroid are a new classification of thyroid tumors in the World Health Organization (2017). Only a few cases of evaluating the value of ultrasound combined with pathological examination in diagnosing follicular tumors with uncertain malignant potential of the thyroid have been reported in recent decades.

**Patient concerns:**

A retrospective analysis was performed on 18 patients with follicular tumors with uncertain thyroid malignant potential who underwent preoperative ultrasonography and were confirmed by operation and pathology in our hospital from January 2019 to November 2024. Ultrasonic characteristics, histopathology, and immunohistology were recorded.

**Diagnoses:**

The patient was pathologically diagnosed as follicular tumor of uncertain malignant potential.

**Interventions:**

All the 18 patients underwent ultrasound, surgical treatment and immunohistochemical staining in our hospital.

**Outcomes:**

The patients underwent surgery. There was no complication or recurrence.

**Lessons:**

This case series indicates that the ultrasonographic manifestations of follicular tumors with uncertain thyroid malignant potential were regular and well-defined hypoechoic thyroid masses with an elastic grade of 2. The pathological characteristics were positive expression of P53, TG, CD56, Ki67, TTF-1, and Gal-3 mutations in most lesions.

## 1. Introduction

In recent years, the incidence of thyroid nodule lesions in China has shown an increasing trend. Papillary thyroid carcinoma (PTC) is the most common histological subtype of thyroid cancer, followed by follicular thyroid cancer.^[1]^ In the 2017 edition of the World Health Organization classification of thyroid tumors, “borderline thyroid follicular tumor” was newly added, including^[2–4]^ well-differentiated tumor of uncertain potential, follicular tumor of uncertain potential (follicular tumor of uncertain potential) malignant potential (FT-UMP), and noninvasive follicular thyroid neoplasm with papillary-like nuclear features).

FT-UMP is a rare thyroid tumor, and its pathological characteristics are as follows^[4–7]^: the cancer is wholly composed of follicles without papillary structure; the tumor has round nuclei, lacking the nuclear characteristics of papillary carcinoma (enlarged, elongated, crowded, or overlapping nuclei; the atomic envelope is irregular, with nuclear sulci or pseudo inclusion bodies visible; and nuclear transparent or ground glass); and the tumor envelope is complete or well delimited, but there is uncertain envelope invasion or vascular invasion. The biological behavior of these tumors is so inert that additional radioiodine therapy is not required. Therefore, accurate diagnosis of such tumors before surgery can not only reduce the overtreatment of patients but also reduce the psychological burden of patients.

FT-UMP research mainly focuses on its clinical and pathological features, but there are few reports on its ultrasonographic findings. This paper systematically discusses the clinical and ultrasonic pathological features, diagnosis, and differential diagnosis of the tumor from clinical features, ultrasonic manifestations, pathomorphology, and immunohistochemical staining results.

## 2. Materials and methods

### 2.1. Research object

From January 2019 to November 2024, 18 patients with complete clinical, ultrasound (US), and pathological data were confirmed as FT-UMP by operation in our hospital, including 16 females and 2 males, aged from 25 to 68 years old, with an average age of 48.61 ± 12.64 years. Six cases were located in the left lobe (33.3%), 5 cases were located in the right lobe (27.8%), and 7 cases were located in the isthmus (38.9%). The ratio of males to females was about 1:8; 7 cases were younger than 45 years old, accounting for 38.9%, and 11 cases were older than 45 years old, accounting for 61.1% (Table 1). This study was examined and approved by the ethics committee of our hospital. All the subjects in the study informed consent to this study and signed the informed consent form.

**Table 1:** Clinical characteristics of patients with FT-UMP.

Serial No.	Age (yr)	Gender	Maximum diameter of nodules (cm)	Nodular site	Tg(3.5–77 ng/mL)	Merging Hashimoto thyroiditis	TSH (1.150–131.000 ng/mL)	Free T3 (3.53–7.37 Pmol/L)	Free T4 (7.98–16.02 Pmol/L)
1	60	F	11.6	Right lobe	>481.00	No	2.12	7.95	10.98
2	56	F	2.7	Isthmus	66.33	No	3.06	6.64	10.59
3	56	F	1.5	Left lobe	5.80	Yes	1.80	4.71	12.47
4	34	F	1.3	Isthmus	39.81	No	4.10	7.81	14.78
5	42	F	1.4	Isthmus	12.170	Yes	1.54	4.48	9.27
6	58	F	3.8	Isthmus	>481.00	No	1.36	5.62	11.90
7	31	M	4.1	Right lobe	112.790	No	1.54	5.93	10.88
8	25	F	3.1	Isthmus	13.030	No	3.32	5.59	8.89
9	40	F	5.6	Left lobe	>481.00	No	1.65	5.62	10.41
10	54	F	4.2	Left lobe	>505.00	No	2.34	5.66	8.94
11	58	F	3.0	Isthmus	>481.00	No	4.42	5.17	7.83
12	50	F	0.8	Isthmus	0.650	Yes	5.85	5.49	11.58
13	57	F	0.55	Right lobe	>481.00	No	5.45	4.56	10.34
14	41	F	1.1	Left lobe	0.510	Yes	3.15	3.84	9.28
15	63	F	1.5	Right lobe	0.470	No	1.54	5.47	9.92
16	30	M	2.9	Left lobe	379.080	No	2.67	6.34	10.17
17	52	F	0.2	Left lobe	11.16	No	2.55	4.62	11.08
18	68	F	4	Right lobe	15.08	No	3.56	5.83	10.76

F = female, FT-UMP = follicular tumor of uncertain malignant potential, M = male.

Inclusion criteria were as follows: ultrasonography within 1 month before thyroid operation; conscious patients who voluntarily participated in the study and signed informed consent; surgical treatment of thyroid tumors in our hospital; and pathologically confirmed FT-UMP.

Exclusion criteria were as follows: pregnant or lactating women; patients with severe heart, liver, kidney, and other organ complications and blood system diseases; unclear US images; and those who did not perform surgery because of primary diseases.

## 3. Instruments and methods

### 3.1. US inspection method

The instrument and equipment are a Q5Philipshealthcare (USA) color Doppler US diagnostic instrument, a linear array high-frequency probe with a probe frequency of 5–12 MHz, and a “Thyroid” condition set by the tool. During the examination, the patient took the supine position, removed the pillow, and tilted the neck slightly, exposing the neck as far as possible. The thyroid was examined by 2-dimensional US, and the probe was placed under the thyroid cartilage for cross-sectional and longitudinal examination. Determine the location, diameter, boundary, internal echo, shape, and number of lesions, and observe the superficial and deep parathyroid muscles, trachea, esophagus, and large blood vessels. Then, the distribution and richness of blood flow in and around the nodules were observed by color Doppler US.

Elastic ultrasonography: Select the section with the best display of nodules, fix the probe, and switch to the side-by-side mode of flexible imaging; the sampling frame is larger than the scope of nodules (generally 2–3 times the size of nodules); try to include part of normal tissues when nodules are large, and continuously observe the elastic imaging images. During the operation, the patient was asked to hold his breath intermittently, the probe was kept stable, and the observation section remained unchanged as far as possible to obtain durable elastic imaging images. Static and dynamic images are recorded in DICOM format on the device’s built-in hard disk.

### 3.2. Image analysis method

The imaging data of the subjects were blindly analyzed by ≥2 attending physicians in the US department of our hospital; 2 attending physicians who have been engaged in US diagnosis for 10 years obtained the original US images and conducted a double-blind analysis of the images, whether the nodules were in contact with the thyroid capsule and whether the adjacent organs were invaded. The ultrasonic images focus on the location, size, boundary, shape, internal echo, involved range, elastic grading, and other signs of the nodules. When there is disagreement, the final results are based on the surgical records and pathological consequences.

### 3.3. Pathological and immunohistochemical examination

All the 18 patients were diagnosed as FT-UMP by histopathological and immunohistochemical analysis. The diagnostic criteria were as follows^[8–10]^: the tumor was composed of well-differentiated follicular cells, no nuclear characteristics of papillary carcinoma, no prominent atypia, and no pathological mitosis; the cancer had a complete capsule and a clear boundary. The capsule is often thick or uneven; and suspected capsule/vascular infiltration is necessary for its diagnosis. Among the 18 patients, 16 underwent fine-needle aspiration biopsy before surgery.

### 3.4. Statistical analysis

The data in this paper are statistically analyzed by SPSS 20.0 statistical software, and the measurement data are expressed by mean ± standard deviation ($\bar{x}\pm s$).

## 4. Result

### 4.1. Clinical characteristics of patients with FT-UMP

All 18 patients with FT-UMP were confirmed by operation and pathology. Six cases were located in the left lobe, 5 in the right lobe, and 7 in the isthmus. The maximum diameter of the tumor was 0.2–11.6 cm, with an average of 2.96 ± 2.63 cm, of which 3 cases were <1 cm and 15 cases were >1 cm. T3 was slightly increased in 2 cases, Tg was abnormally increased in 8 cases, and Hashimoto thyroiditis was found in 4 cases. These 18 cases were followed up, and there were no deaths, recurrences, cervical lymph nodes, or distant metastases, and the overall prognosis was good.

### 4.2. Ultrasonic feature diagnosis

**Figure 1.**
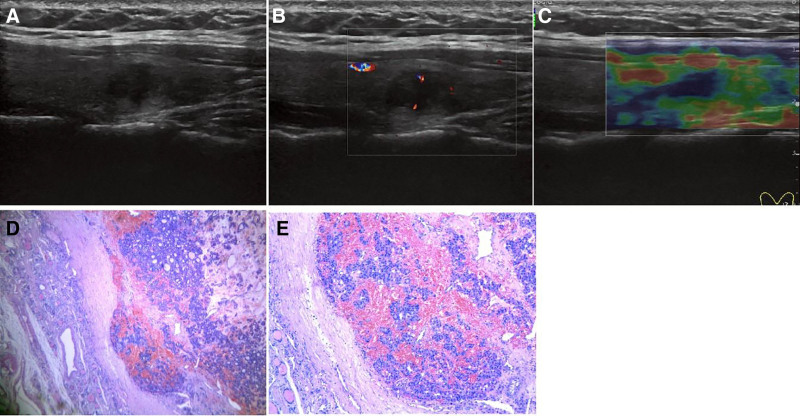
A 63-year-old female patient presented with a follicular tumor of uncertain malignant potential in the left lobe of the thyroid gland. (A) The ultrasound longitudinal incision showed a less regular hypoechoic mass in the left lobe of the thyroid with a clear boundary and less uniform internal echo. (B) Color Doppler flow imaging showed a small number of blood flow signals around and inside. (C) Elasticity score: 2. (D and E) Suspected capsular infiltration of follicular tumor of uncertain malignant potential hematoxylin and eosin (4×10).

The ultrasonic features of FT-UMP are shown in Table 2. Sixteen cases were oval, and 2 patients were irregular. The boundary was apparent in 11 cases; the capsule was intact in 11 cases; the border was unclear in 3 instances, undefined in 4 patients, and cervical lymph node metastasis in 3 cases. Twelve cases were hypoechoic, 3 cases were isoechoic, and 3 cases were very hypoechoic. The elasticity score was 3 in 7 patients and 2 in 11 patients (Fig. 1).

**Table 2:** Ultrasonic characteristics of patients with FT-UMP.

Serial No.	Age (yr)	Internal echo	Boundary	Form	Internal calcification	Helo sign	Aspect ratio > 1	Internal blood flow	Peripheral blood flow	Elastic score	Cervical lymph node metastasis
1	60	Isoechoic	Lack of clarity	Oval	Yes	No	No	Rich	Small amount	2	No
2	56	Isoechoic	Clear	Oval	No	Yes	No	Small amount	Small amount	2	No
3	56	Hypoechoic	Clear	Oval	Yes	No	No	Rich	Encircling	2	No
4	34	Extremely low echo	Lack of clarity	Oval	No	No	No	Small amount	Small amount	3	No
5	42	Hypoechoic	Clear	Oval	No	No	No	Small amount	Small amount	2	Yes
6	58	Hypoechoic	Clear	Oval	Yes	No	No	Small amount	Small amount	3	No
7	31	Hypoechoic	Clear	Oval	No	No	No	Small amount	Small amount	2	Yes
8	25	Hypoechoic	Clear	Oval	Yes	No	No	Small amount	Arc-shaped	2	No
9	40	Hypoechoic	Unclear	Irregular	No	No	No	Small amount	Small amount	2	No
10	54	Hypoechoic	Clear	Oval	No	No	No	Small amount	Arc-shaped	2	No
11	58	Hypoechoic	Clear	Oval	Yes	No	No	Rich	Arc-shaped	2	No
12	50	Hypoechoic	Clear	Oval	No	Yes	No	Rich	Arc-shaped	3	No
13	57	Extremely low echo	Unclear	Oval	No	No	No	Small amount	Encircling	3	No
14	41	Hypoechoic	Unclear	Oval	Yes	No	No	Rich	Small amount	3	No
15	63	Extremely low echo	Lack of clarity	Irregular	Yes	No	No	No	Small amount	3	No
16	30	Isoechoic	Clear	Oval	No	Yes	No	Rich	Encircling	2	Yes
17	52	Hypoechoic	Lack of clarity	Oval	No	No	No	No	No	3	No
18	68	Hypoechoic	Clear	Oval	No	No	No	Small amount	Arc-shaped	2	No

FT-UMP = follicular tumor of uncertain malignant potential.

The ultrasonographic features of FT-UMP were hypoechoic thyroid nodules with regular shape and clear boundary, complete capsule, no compression of adjacent tissue, no local invasion, and an elasticity score of 2.

### 4.3. Histopathological and immunohistochemical diagnosis

Fine-needle aspiration biopsy was performed in 16 of the 18 patients. Twelve patients were diagnosed with PTC, 2 were diagnosed with atypical lesions, and 2 were dissatisfied with the samples and could not be diagnosed. In the hematoxylin and eosin sections of 18 patients with FT-UMP, the pathological morphology showed that the tumor was composed of encapsulated follicles without the nuclear features of PTC, and there was suspected capsule/vascular infiltration. In immunochemical staining, the positive rate of CD56, Ki67, TTF-1, and Gal-3 was more than 50%, including 10 cases of Gal-3 (+), 13 cases of CD56 (+), 15 cases of Ki67 (+), 13 cases of TTF-1 (+), 14 cases of TG (+), and 12 cases of p53 (+) (Table 3).

**Table 3:** Pathological characteristics of immunohistochemical examination in patients with FT-UMP.

	Age (yr)	Gal-3	CD56	Ki67	TTF-1	TG	P53
1	60	−	+	+2%	+	+	−
2	56	−	+	−	−	+	−
3	56	+	+	+2%	+	+	+5%
4	34	+	+	+5%	+	+	+10%
5	42	+	−	+1%	+	+	+
6	58	+	−	+1%	−	+	+35%
7	31	+	+	+5%	+	−	+
8	25	−	−	+5%	+	+	+20%
9	40	−	+	−	−	+	−
10	54	−	+	+2%	+	−	+30%
11	58	+	+	+5%	+	−	+10%
12	50	−	+	−	+	+	−
13	57	+	+	+2%	+	+	+5%
14	41	+	+	+3%	+	+	+10%
15	63	+	−	+8%	+	+	+25%
16	30	−	+	+1%	−	+	−
17	52	+	−	+2%	−	−	+
18	68	−	+	+8%	+	+	−

FT-UMP = follicular tumor of uncertain malignant potential.

## 5. Discussion

In 2000, Williams put forward the concept of FT-UMP, which is used to name the lesions of encapsulated follicular growth patterns that are morphologically noninvasive, suspected infiltration, and have the characteristics of uncertain PTC nuclei. The incidence of this kind of tumor is low, and it has been reported that^[10–13]^ FT-UMP accounts for 5.6% of PTC. Although FT-UMP is not invasive in most cases, it is occasionally reported that^[14–16]^ patients diagnosed with FT-UMP for the first time have the possibility of recurrence and metastasis to thyroid follicular carcinoma, indicating that a small number of FT-UMP have malignant potential. Therefore, it is of great clinical significance to diagnose FT-UMP before operation.

The US can be used as the first choice for thyroid tumor imaging with its nonradiation, painless, and real-time dynamic advantages. The US can provide high spatial resolution and display the typical thyroid capsule. However, there are few reports on FT-UMP diagnosis in the US. In this study, 18 cases of FT-UMP had a wide range of ages, ranging from 25 to 68 years, with an average of 48.61 ± 12.64 years, with a maximum diameter of 0.2–11.6 cm and an average of 2.96 ± 2.63 cm. All lesions had clear boundaries and intact capsules. Tg was significantly increased in 8 patients with FT-UMP. Three cases of FT-UMP with cervical lymph node metastasis may be associated with PTC simultaneously.

In this study, 16 cases of US were diagnosed with PTC, 1 patient was diagnosed with benign thyroid nodules, and 1 case of US was diagnosed with subacute thyroiditis. In this study, the ultrasonic manifestations of 18 instances of FT-UMP were mainly hypoechoic or very hypoechoic, with regular nodules, clear boundaries, uniform or no halo ring thickness, and a few internal calcification points. Typical ultrasonic signs of PTC are as follows^[13,14]^: irregular shape, unclear boundary or incomplete halo, uneven internal hypoechoic or polar echo, accompanied mainly by sand-like intense echo calcification, aspect ratio > 1, easy to distinguish from FT-UMP. Sixteen cases were identified through US, likely due to the unusual US presentation in these instances, coupled with the relative rarity of FT-UMP, which may have led US radiologists to overlook this diagnosis. Among them, 1 case was diagnosed as subacute thyroiditis by US. Misdiagnosis occurred because this case presented with a low echo area of about 22×20 mm, unclear boundary, irregular shape, and a small amount of internal blood flow signal, consistent with typical ultrasonic manifestations of subacute thyroiditis. One patient was misdiagnosed as having a benign nodule, probably because the nodule contained cystic components.

Capsular/vascular infiltration is a necessary condition for the pathological diagnosis of FT-UMP. Currently, it is considered that the suspicious capsular infiltration modes are as follows^[17–21]^: the capsular membrane of fibrous connective tissue in a broad basal range of tumor cells; the tumor cells connected to the tumor body showed mushroom infiltration of the capsular membrane but did not penetrate; the tumor body at the capsular junction infiltrated but did not penetrate the capsular membrane in the way of budding: and thyroid follicles exist between thickened fibrous envelopes, and the long axis is perpendicular to the envelope. The suspicious ways of vascular infiltration include lack of endothelial cell coating in the vascular space and tumor cell nests in the fibrous connective tissue in contact with blood vessels, which cannot be identified as early vascular infiltration or pure tumor cell nests and blood vessels. In this study, hematoxylin and eosin sections of 18 FT-UMP patients showed suspicious capsule or vascular infiltration in pathological morphology. None of the 16 cases that underwent fine-needle aspiration were diagnosed with FT-UMP, mainly because the diagnosis of FT-UMP depends on capsule/vascular infiltration, so fine-needle aspiration biopsy has great limitations in differentiating FT-UMP from PTC.

In this study, 10 FT-UMP patients showed positive Gal-3, 13 positive CD56, 15 weakly positive Ki67, 13 positive TTF-1, 14 positive TG, and 12 favorable P53 mutations (+). Gal-3, a member of the β-galactosidase binding protein family with a molecular mass of 31 ku, mainly exists in the cytoplasm and the membrane and nucleus. It has been proved by studies^[21,22]^ that Gal-3 can be used as a phenotypic feature of thyroid malignant tumors, especially PTC, and as a marker of PTC, with high sensitivity and specificity. Gal-3 is associated with the progression and poor prognosis of thyroid tumors and is one of the driving factors of tumor malignant transformation and has high accuracy in distinguishing benign and malignant tumors.^[23,24]^ CD56 is a nerve cell adhesion molecule generally expressed in natural killer cells, activated T cells, and nerve and muscle tissues, and its expression regulates the motility and migration of tumor cells. It has been reported^[25–27]^ that CD56 is highly expressed in normal thyroid, benign or malignant thyroid follicular tumors but not expressed or lowly expressed in PTC tissues. In this study, 6 cases expressed Gal-3 and CD56 simultaneously, which may indicate that FT-UMP is a type of PTC with a malignant potential. P53 is a tumor suppressor gene. More than 50% of all malignant tumors have mutations in this gene, indicating that the change of this gene is likely to be the primary pathogenesis of human tumors. Like all other tumor suppressor factors, P53 plays a role in slowing down or monitoring cell division under normal conditions. After mutation of the P53 gene, due to the change of its spatial configuration, the regulation of cell growth, apoptosis, and DNA repair is lost, and the P53 gene changes from a tumor suppressor gene to an oncogene. In our study, 12 FT-UMP patients showed P53 mutation (+), and 9 showed a mutation percentage ≥ 5%. However, no relevant reports on P53 mutation (+) in FT-UMP patients were found in previous studies, which may indicate that these 12 FT-UMP patients have more substantial malignant potential. Previous studies^[21,22,25–27]^ showed that Ki67 had low or no FT-UMP expression. In this study, 15 cases were weakly positive for Ki67, which marked cells in the local proliferation cycle. The higher the favorable rate, the higher the proportion of tumor cells in the growth stage, the faster the tumor growth, and the more malignant and aggressive the tumor. Among the 15 cases showing weak Ki67 cheerful, 12 patients were accompanied by P53 mutation (+), indicating that these 12 cases of FT-UMP may have more substantial malignant potential.

This study still has the following shortcomings: as a retrospective study, there may be selectivity bias, which may have an impact on statistics; as a single-center study with a small sample size, we will conduct a multi-center study and increase the sample size in further studies; and we will conduct multi-parameter US diagnosis of FT-UMP in the later stage.

In conclusion, US is used as the primary screening method, combined with peripheral blood examination and histopathologic examination for all sampling, immunohistochemical analysis, and molecular detection of the capsule, to improve FT-UMP diagnosis and provide a more reliable basis for clinical treatment.

## Acknowledgments

The corresponding author would like to express his sincere appreciation to all individuals who contributed to the completion of this research project. Their support, guidance, and expertise were invaluable throughout the process.

## Author contributions

**Conceptualization:** Xian Wang.

**Writing – original draft:** Xian Wang.

**Investigation:** Hui Zhou, Donggang Pan.

**Software:** Muhammad Asad Iqbal.

**Writing – review & editing:** Muhammad Asad Iqbal, Nida Fatima Moazzam.

**Resources:** Junbo Zuo.

**Funding acquisition:** Jin Zhang.

**Supervision:** Hui Sun.
